# Systemic Therapy for Metastatic Triple Negative Breast Cancer: Current Treatments and Future Directions

**DOI:** 10.3390/cancers15153801

**Published:** 2023-07-26

**Authors:** Laura Morrison, Alicia Okines

**Affiliations:** Breast Unit, The Royal Marsden NHS Foundation Trust, London SW3 6JJ, UK

**Keywords:** metastatic breast cancer, breast neoplasms, TNBC, triple negative breast cancer, systemic therapy, immunotherapy, ADCs, antibody-drug conjugates

## Abstract

**Simple Summary:**

Triple negative breast cancer, a subtype of breast cancer that does not respond to hormone therapy, is typically more aggressive and affects younger patients. Treatment has traditionally been limited to chemotherapy, with poor survival for patients with advanced disease. More recently a deeper understanding of the subtypes of triple negative breast cancer have allowed the development of several new treatments, including immunotherapy and targeted therapies, which have shown promising results in clinical trials. These developments are expanding the treatment options available for this group of patients and leading to much needed improvements in survival. In this review, we summarise the recent developments and discuss the future treatment of triple negative breast cancer.

**Abstract:**

Until recently, despite its heterogenous biology, metastatic triple negative breast cancer (TNBC) was treated as a single entity, with successive lines of palliative chemotherapy being the only systemic option. Significant gene expression studies have demonstrated the diversity of TNBC, but effective differential targeting of the four main (Basal-like 1 and 2, mesenchymal and luminal androgen receptor) molecular sub-types has largely eluded researchers. The introduction of immunotherapy, currently useful only for patients with PD-L1 positive cancers, led to the stratification of first-line therapy using this immunohistochemical biomarker. Germline *BRCA* gene mutations can also be targeted with PARP inhibitors in both the adjuvant and metastatic settings. In contrast, the benefit of the anti-Trop-2 antibody-drug conjugate (ADC) Sacituzumab govitecan (SG) does not appear confined to patients with tumours expressing high levels of Trop-2, leading to its potential utility for any patient with an estrogen receptor (ER)-negative, HER2-negative advanced breast cancer (ABC). Most recently, low levels of HER2 expression, detected in up to 60% of TNBC, predicts benefit from the potent HER2-directed antibody-drug conjugate trastuzumab deruxtecan (T-DXd), defining an additional treatment option for this sub-group. Regrettably, despite recent advances, the median survival of TNBC continues to lag far behind the approximately 5 years now expected for patients with ER-positive or HER2-positive breast cancers. We review the data supporting immunotherapy, ADCs, and targeted agents in subgroups of patients with TNBC, and current clinical trials that may pave the way to further advances in this challenging disease.

## 1. Introduction

The term triple negative breast cancer (TNBC) encompasses all cancers that do not express the oestrogen, progesterone, or HER2 receptors at targetable levels. Up to 20% of breast cancer diagnoses are triple negative [1], with enrichment in younger patients, those of African-American descent [2,3,4], and known carriers of germline *BRCA1* mutations [5].

Most cases of metastatic TNBC do not occur de novo but as relapses in patients who have been previously treated for early breast cancer (EBC). The majority of patients will have received (neo) adjuvant multi-agent chemotherapy and, increasingly, many will have additionally been exposed to immunotherapy with the anti-PD1 monoclonal antibody, pembrolizumab [6]. For patients relapsing a year or more after completing treatment for EBC, re-challenge with previously received chemotherapy agents, with or without immunotherapy, may be reasonably attempted, but little data exist in this setting. Treatment for patients progressing on or soon after completing EBC treatment inevitably poses a greater challenge given the likelihood of multi-agent resistance in their cancers.

**Figure 1:**
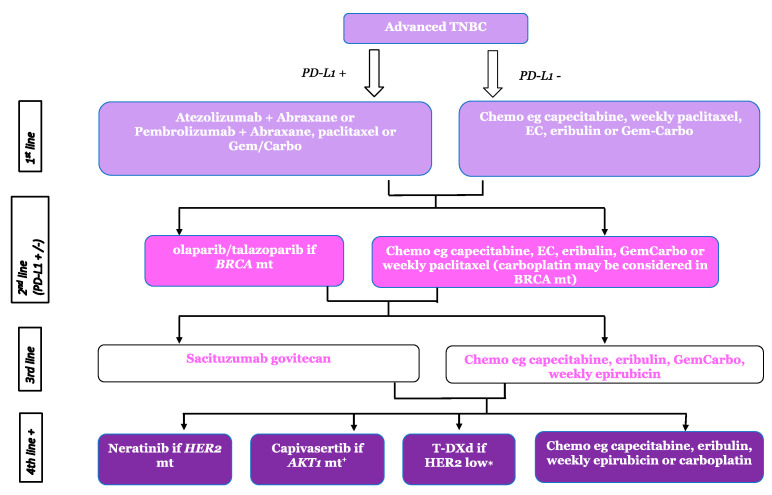
Proposed treatment algorithm for de novo metastatic breast cancer. TNBC = triple negative breast cancer, PDL-1 = programmed death ligand-1, BRCA mt = BRCA mutation, HER2 mt = HER2 mutation. + Currently unlicensed so only available within a clinical trial, * Can also be used in 2nd line in line with the EU license.

**Figure 2:**
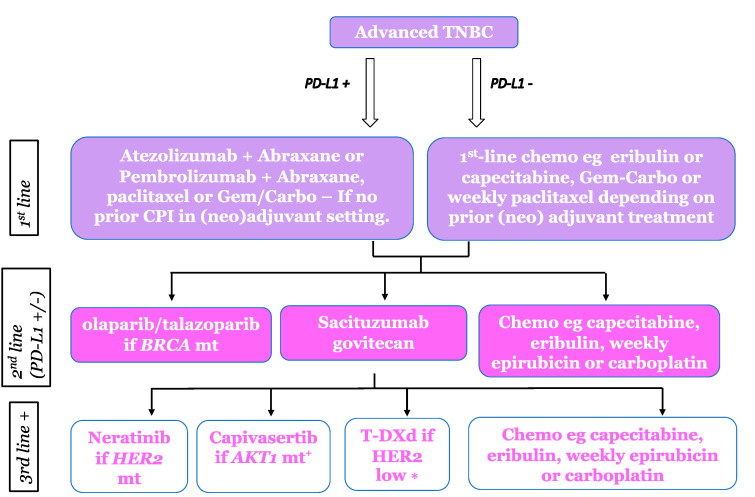
Proposed treatment algorithm for metastatic breast cancer following (neo) adjuvant chemotherapy. AKT mt = AKT mutation. + Currently unlicensed so only available within a clinical trial, * Can also be used in either 2nd line or 1st line if relapsing within 6 months of adjuvant chemotherapy in line with the EU license.

TNBC has traditionally been treated as a homogenous group with no available targeted therapies and a reliance on sequential standard chemotherapies, resulting in poor outcomes relative to oestrogen receptor (ER)-positive or HER2-positive breast cancers. A greater understanding of the heterogeneity of TNBC is becoming more widespread following gene expression profiling studies [7]. While mostly conducted in EBC samples, these studies initially defined six biological subtypes (TNBCtype); the basal-like 1 and 2 (BL-1 & BL-2) groups characterised by high expression of cell cycle and DNA damage response genes; immunomodulatory (IM) cancers; mesenchymal-like and mesenchymal stem-like cancers (M & MSL) enriched for gene expression for epithelial-mesenchymal transition and growth factor pathways; and the luminal androgen receptor (LAR) subtype characterised by androgen receptor signalling [7]. The same group later refined the classification to four sub-types correlating them to clinical behaviour, including chemotherapy responsiveness [8]. Despite this, the TNBCtype-4 classification failed to gain clinical utility and TNBC continued to be treated as a single entity. However, this understanding gives the potential for a more personalised approach to the treatment of advanced TNBC and highlights a pressing need to identify suitable biomarkers to determine benefit from specific therapeutic agents. We have generated a treatment algorithm for both de novo and relapsed metastatic breast cancer, taking into consideration recent advances and new approvals, which will be discussed in this review (Figure 1 and Figure 2).

## 2. Immunotherapy

Immune checkpoint inhibitors (ICIs) have revolutionised the treatment of several solid tumours such as melanoma and lung cancer [9,10]. Historically, breast cancer was expected to be resistant to ICI due to an “immune cold” phenotype [11]. The identification of tumour infiltrating lymphocytes (TILs) and their role in treatment responses [12] as well as genomic evidence for immune responses in TNBC and HER2+ disease [13] renewed interest in this therapeutic strategy for these sub-types of breast cancer.

Initial ICI monotherapy trials were associated with modest response rates of between 5 and 20% in patients who were heavily pre-treated [14], although a small number appeared to have durable benefit with long-term ICI treatment. There was an increasing probability of response in those who were PD-L1 positive [14], which has since been established as a biomarker for the identification of patients with advanced TNBC who are likely to benefit from ICI. Disappointingly, no significant benefit was reported for pembrolizumab monotherapy over standard chemotherapy in previously treated TNBC patients in the Keynote-119 study (KN-119) [15]. The inclusion of patients irrespective of PD-L1 status may have influenced the negative study result, as an exploratory sub-group analysis reported potential benefit from pembrolizumab (ORR 26% compared to 12% with chemotherapy) in the 109/622 randomised patients with a combined positive score (CPS) of 20 or more for PD-L1 expression [16]. This cut-off for CPS score is higher than that required for significant benefit in the first-line setting (≥10) [17]. The choice of chemotherapy in the control arm (platinum was not permitted) also affects the generalisability of the KN-119 data in PD-L1-positive patients. These results have now been largely superseded by data from first-line studies [15].

ICI monotherapy is not currently approved for breast cancer other than within the two FDA tumour agnostic approvals; one for patients with micro satellite instability—high (MSI-H) tumours [18] approved in 2017—and one for those with a high tumour mutation burden (TMB), both based on early phase clinical trials [19,20]. The implications of these approvals for the breast cancer cohort are less certain, as KN-158 included just five breast cancer patients and none were included in KN-016 [20]. Rates of MSI-H are low in breast cancer compared to other solid tumours [21,22], although slightly higher in TNBC compared to other subtypes [23] and the frequency of high TMB in the PD-L1 negative sub-group (who will not have received first-line chemo-immunotherapy) is unknown.

Positive results with ICI in TNBC were finally reported once they were added to a first-line chemotherapy backbone (Table 1) [24]: The IMpassion130 trial was the first study conducted in TNBC to show an overall survival benefit from the addition of ICI to standard first-line chemotherapy [25]. The anti-PD-L1 monoclonal antibody (mAb) atezolizumab improved response rate (RR) (56.0% with ICI vs. 45.9% with placebo), progression-free survival (PFS) (HR 0.62; 95% CI, 0.49–0.78), and overall survival (OS) (HR 0.62; 95% CI, 0.45–0.86) when added to nab-paclitaxel in patients with PDL-1-positive immune cells covering 1% or greater of the tumour area using the companion diagnostic assay (Ventana PD-L1 SP142) [25]. The subsequent IMpassion131 study was expected to confirm benefit from adding atezolizumab to the more widely used paclitaxel; however, no benefit was reported either in the overall study population or in patients with PD-L1-positive cancers [26]. The manufacturer of atezolizumab, Roche, subsequently voluntarily withdrew from their accelerated FDA approval [27], but the combination remains approved by the EMEA and other regulatory bodies. There has yet to be a satisfactory explanation for the conflicting results seen in IMpassion130 and 131 despite in-depth analysis of both studies [28]. The IMpassion132 study (NCT03371017) will evaluate the addition of atezolizumab to first-line chemotherapy, specifically targeting those relapsing within 1 year of completing chemotherapy for EBC who were excluded from both IMpassion130 and 131. However, this will not answer the emerging question regarding the utility of first line immunotherapy in patients who have received (neo-)adjuvant pembrolizumab.

In contrast to atezolizumab, pembrolizumab demonstrated benefit whether added to nab-paclitaxel, paclitaxel, or a gemcitabine-carboplatin doublet (the latter recommended within the study for those relapsing within 6–12 months after (neo) adjuvant therapy) for patients with PD-L1-positive metastatic TNBC defined by a CPS of ≥ 10 using the PD-L1 IHC (immunohistochemical) 22C3 assay [29]. Pembrolizumab significantly improved ORR (52.7% vs. 40.8% in ITT group), median PFS (HR 0.66, CI 0.50–0.88), and median OS (HR 0.73, CI 0.55–0.95) in the PD-L1-positive sub-population, so is a first-line alternative to atezolizumab where both are approved. The outcomes for PD-L1 positive patients in IMpassion-130 and Keynote-355 were similar, so either agent can be effectively used with a nab-paclitaxel backbone where available, subject to positive PD-L1 by the appropriate companion diagnostic. Importantly, concordance of the two assays when the CPS cut-off of ≥10 is applied is approximately 75%, so more patients can benefit from ICI if both tests are undertaken: [30] In a post-hoc analysis of IMpassion130 patients whose tissue was tested using multiple PD-L1 assays, the PD-L1+ prevalence was 46.4% with the SP142 assay and 52.9% with the 22C3 assay (using the CPS ≥ 10 cut-off) [30], with almost all cases that were SP142 positive also testing positive using the 22C3 antibody, and the greatest benefit from atezolizumab noted in the “double positive” cases.

Metastatic or primary breast tissue can be used for PD-L1 testing, depending upon availability, as there is strong concordance between PD-L1 expression in primary and metastatic samples (92.6%) [31], although primary tissue is slightly more likely to be PD-L1 positive [32].

Of interest, those patients with higher PD-L1+ expression in metastatic samples appeared to derive the greatest benefit from treatment in a small analysis of IMpassion130 patients [30]. This was perhaps because this more closely reflects the immunological status of their cancer at the time of treatment. For patients with metastatic biopsies, the site of disease sampled is important as primary breast tissue and axillary nodes have a higher rate of PD-L1 expression than liver metastases, which are relatively immunologically cold [32,33].

**Table 1:** Results from randomised phase 3 trials of immunotherapy in TNBC (triple negative breast cancer).

Trial Name and Sample Size	Treatment Arms	Response Rate PD-L1+ (ITT)	Median PFS (Months) PD-L1+ (ITT)	Median OS (Months) PD-L1+ (ITT)
First Line (combination with chemotherapy)				
IMpassion130 [25] *n* = 369 PD-L1+ (ITT *n* = 902)	Nab-paclitaxel + placebo	42.6% (45.9%)	5.0 (5.5)	18 (18.7)
	Nab-paclitaxel + atezolizumab	58.9% (56.0%)	7.5 (7.2)	25 (21)
IMpassion131 [26] *n* = 292 PD-L1+ (ITT *n* = 651)	Paclitaxel + placebo	55% (54%)	5.7 (5.6)	28.3 (22.8)
	Paclitaxel + atezolizumab	63% (47%)	6.0 (5.7)	22.1 (19.2)
Keynote-355 [29] *n* = 323 PD-L1+ (ITT *n* = 847)	Chemo (paclitaxel, nab-paclitaxel or Gem/carbo) + placebo	55% (47%)	5.6 (5.6)	16.1 (15.5)
	Chemo + pembrolizumab	63% (54%)	9.7 (7.5)	23.0 (17.2)
≥2nd line (monotherapy)				
Keynote-119 [15] *n* = 194 PD-L1+ (ITT *n* = 622)	Chemo (capecitabine, eribulin, gemcitabine or vinorelbine)	9.2% (10.6%)	3.4 (3.3)	11.6 (10.8)
	Pembrolizumab	17.7% (9.6%)	2.1 (2.1)	12.1 (9.9)
≤2nd line—single arm				
ENHANCE 1 [34] *n* = 74 PD-L1+ (ITT *n* = 167)	Pembrolizumab + Eribulin (single arm)	28.4% (23.4%)	6.1 in 1st line, 4.1 in 2nd line (4.1)	21.0 in 1st line; 14.0 in 2nd line (16.1)

PD-L1+ = PD-L1 positive ≥ 1% IC by SP142 assay for atezolizumab studies or CPS score ≥ 10 for pembrolizumab studies.

Regrettably, acquired resistance develops in most patients treated with ICI, although a small proportion will benefit from a long-term remission. Combination regimens, including ICI with radiotherapy and/or PARP inhibitors (NCT04683679, NCT04837209), oncolytic viruses (NCT05081492), and vaccines (NCT03606967), are being explored to attempt to overcome primary and acquired ICI resistance.

## 3. Antibody-Drug Conjugates

Three antibody-drug conjugates (ADCs) have demonstrated clinical utility in TNBC to date with two approved for clinical use (Table 2 for summary). ADCs deliver a chemotherapy “payload” linked to an antibody against a specific antigen such as HER2 or Trop2. This abrogates the risk of systemic toxicities from the cytotoxic, which is delivered specifically to cancer cells expressing the relevant antigen. Increasing the ratio of payload to antibody and the use of cleavable linkers both enables delivery of the payload to adjacent cells that do not express the target antigen (known as the bystander effect) but also inevitably increases the systemic toxicity.

### 3.1. Sacituzumab Govitecan (SG)

Trop 2 is a transmembrane protein expressed by most TNBCs and, although not itself a drug target, has been successfully used as a means of delivering a topoisomerase 1 inhibitor, SN38, to Trop 2 expressing tumour cells. A phase 2 study in which patients with epithelial tumours including 144 participants (29% of study participants) with TNBC [45] showed a promising 33.3% ORR in the TNBC cohort, leading to accelerated FDA approval [46]. Confirmatory phase 3 data followed from the ASCENT trial [35] within which patients with advanced TNBC who had received two or more lines of therapy for ABC were randomized to SG or single-agent chemotherapy of the physician’s choice (eribulin, vinorelbine, capecitabine or gemcitabine). SG significantly improved objective RR (35% vs. 5%), PFS (HR 0.43; 95% CI, 0.35 to 0.54), and OS (HR 0.51; 95% CI, 0.41 to 0.62) [35]. Tumour Trop 2 levels did not significantly correlate with benefit from the ADC and no heterogeneity of effect was seen across pre-specified subgroups. In a subsequent analysis of the 61 patients with stable brain metastases who were included in the study but were not part of the primary endpoint population, benefit was uncertain, with few responses (3% vs. 0%) [47]. There was a numerically longer median PFS (2.8 vs. 1.6 months) but no significant improvement in OS (6.8 vs. 7.5 months) [47]. These results are perhaps surprising given that the cytotoxic component of SG (SN-38) is able to cross the blood–brain barrier (BBB) [48,49]. A study is underway to confirm the ability of SG to cross the BBB in TNBC patients (NCT03995706) and the efficacy of SG will be prospectively evaluated in a phase 2 study of SG monotherapy in patients with TNBC and progressive brain disease (NCT 04647916). Two phase 3 studies are underway in the first-line setting, evaluating SG monotherapy for PD-L1 negative TNBC (NCT05382299, ASCENT-03) and SG added to pembrolizumab for PD-L1-positive TNBC (NCT05382286 ASCENT-04). A further study will evaluate adjuvant SG combined with pembrolizumab for patients with residual TNBC after neo-adjuvant chemotherapy (NCT05633654, ASCENT-05).

### 3.2. Trastuzumab Deruxtecan (T-DXd)

The highly potent anti-HER2 ADC T-DXd has shown unprecedented activity in previously treated HER2-positive ABC [50]. Activity was also reported in patients with lower levels of HER2 expression (1+ or 2+ by immunohistochemistry without evidence of elevated gene copy number) [51,52], which led to the DESTINY-Breast04 study [36] in which 557 “HER2 low” patients, including 63 with ER-negative disease (i.e., triple negative), were randomised to T-DXd or chemotherapy of the physician’s choice. The study met its primary endpoint reporting significant prolongation of median PFS in the ER-positive cohort but also demonstrated useful efficacy in the ER-negative patients (median PFS 8.5 vs. 2.9 months, HR 0.46, 95% CI 0.24–0.89) [36]. Although the TNBC cohort was small, this study has defined an effective treatment for the up to 60% of patients with ER-negative/HER2 low advanced breast cancer [53] and provides an alternative or perhaps additional option to SG. A degree of cross-resistance between the two ADCs is to be expected given the similar payloads; however, the sequential use of these agents may be feasible, as recently demonstrated in the HER2+ population [54]. While the most common grade 3 toxicities in DESTINY-breast04 were neutropenia (13.7%), anaemia (8%), and fatigue (8%), interstitial lung disease or pneumonitis occurred in 12% (*n* = 45) of participants in the experimental arm versus <1% in the SOC (standard of care) arm. While the majority of these were grade 1 or 2, three patients died from this complication (0.8%). There were also 17 patients (4.6%) in the T-DXd arm who developed left ventricular dysfunction, including two grade 3 events. Care must therefore be taken when selecting patients for T-DXd, and close monitoring is essential to detect and treat changes in cardiac and respiratory function as early as possible.

### 3.3. Datopotamab Deruxtecan (Dato-DXd)

A pan-tumour phase 1 study (TROPION-PanTumor01) evaluated this ADC, which, like SG, targets Trop 2 but, like T-DXd, has a deruxtecan payload [55]. Amongst the 44 patients with TNBC, an encouraging 32% ORR was reported [56]. Patients in TROPION-01 had received a median of 3 prior lines of chemotherapy and 30% had received a prior topoisomerase 1-based ADC or an anti-TROP2 ADC. Due to the potential for cross resistance occurring between drugs with the same chemotherapy payload, an analysis of the topoisomerase 1 naïve patients was conducted and reported an improved ORR of 44% compared to 32% seen in all patients. These data suggest a decrement in benefit for patients with prior Topisomerase-1 inhibitor exposure. Although toxicity was generally manageable in this small study and mainly included nausea, vomiting, and fatigue, mucositis occurred in 53% of participants, which was grade 3 in 9%, requiring proactive management from initiation of treatment. Importantly, significant rates of pneumonitis, a key toxicity with T-DXd, have not been reported with Dato-DXd, suggesting that this is mediated via delivery of the payload to HER2-expressing cells. Following these promising results, Dato-DXd is being investigated in the phase 3 TROPION-02 study as a first-line treatment for patients with TNBC who are ineligible for ICI (NCT05374512).

The combination of Dato-DXd with the anti-PD-L1 antibody durvalumab is being evaluated in the first-line setting in the BEGONIA trial, with early results showing an encouraging RR of 74% in 27 patients after a median follow up on 3.9 months [57]. Study guidelines have been amended to improve the management of stomatitis, for which 14% of participants required a dose reduction of Dato-DXd.

### 3.4. Patritumab Deruxtecan

This ADC targets the HER3 receptor delivering the same deruxtecan payload. Fifty-three patients with heavily pre-treated TNBC were included in a phase 1/2 study, which reported a promising 22.6% ORR in this sub-group, median PFS 5.5 months (95% CI 3.9–6.8 months), and median OS of 14.6 months (95% CI 11.2–17.2 months) [58]. ILD (interstitial lung disease) occurred in 6.6%, including one death. The results of one cohort (A) of a follow-up phase 2 study included 19 patients with TNBC treated with patritumab following 1–3 prior lines of chemotherapy and reported efficacy across all levels of HER3 expression [59].

### 3.5. Ladiratuzumab Vedotin (LV) (SGN-LIV1A)

This ADC targets a LIV-1 zinc transporter, expressed by the majority of TNBC [60,61], delivering a microtubule disrupting agent, monomethyl auristatin E (MMAE), a highly potent synthetic analogue of dolastatin 10, to the tumour. Pre-clinical models demonstrated the anti-tumour activity of this compound in both breast cancer-derived cell lines and patient-derived xenografts [60], with a phase 1 study subsequently reporting an encouraging 32% RR in women with pre-treated TNBC unselected by LIV-1 expression [62]. However, the PFS was less impressive at 11.3 weeks, and a further phase 1 study with fractionated weekly dosing in patients with LIV-1-positive TNBC is underway to determine whether the inclusion of a biomarker-selected cohort improves the duration of response (NCT01969643). Updated results show an ORR of 28% in the second line [63] and a tolerable safety profile, with the most common grade 3 AEs (adverse events) being neutropenia, fatigue, hyperglycaemia, hypokalaemia, and hypophosphataemia [63].

Pre-clinical data suggest that LV can induce immunogenic cell death and enhance the immune response to the tumour [64]. The induction of an immune response-related gene expression profile following LV administration within the SGNLVA-001 [62] study provides further evidence for the immune modulating effect of this ADC [65]. As a result of these findings LV, in combination with the anti-PD-L1 antibody pembrolizumab is being investigated in a phase 1/2 study (NCT03310957) for the first-line treatment of TNBC.

### 3.6. Disitamab Vedotin (DV)

DV (RC48) is a HER2-directed ADC that, like LV, has an MMAE payload with a cleavable linker [66]. Preliminary investigation in a phase 1 study included patients with both HER2-positive and HER2-low ABC and demonstrated an ORR of 39.6% (95% CI: 25.8%, 54.7%) and median PFS of 5.7 months (95% CI: 4.1, 8.3 months) in the HER2 low cohort [67]. Further evaluation of DV is underway in previously treated HER2 low ABC including TNBC (NCT05831878).

### 3.7. Enfortumab Vedotin (EV)

This ADC targets a transmembrane protein involved in cell adhesion, Nectin-4, expressed in 78% of breast cancers and more common in cancers of basal subtype [68,69]. Its expression appears to correlate with a worse outcome [69], and, like LV, it delivers a microtubule disrupting MMAE payload. A phase 2 study, which will include a cohort of approximately 40 patients with previously treated TNBC, is currently recruiting (NCT04225117). This agent has been approved in metastatic urothelial cancer based on a phase 3 trial that did not require NECTIN-4 expression for study entry [70]. However, the results of a subsequent study suggested that NECTIN-4 levels may fall with metastatic spread and that low levels are associated with resistance to EV [68].

### 3.8. ADC Summary

The development and availability of ADCs are changing the treatment paradigm for TNBC and provide promising targeted therapy for this group of patients. There are a significant number of ongoing clinical trials with the ADCs described here as well as novel ADCs (Table 3), so further changes to the treatment paradigm are expected in the future.

## 4. Targeted Agents

A number of specific vulnerabilities in the genetic and cellular processes of the cancer cell have also been identified, leading to the development of a several clinical trials involving “targeted agents” (Table 2 for a summary of clinical trials).

### 4.1. PARP Inhibitors

Mutations in the DNA repair genes *BRCA1* and *BRCA2* are associated with a substantially increased lifetime risk of breast cancer, and mutations in *BRCA1* are particularly associated with the development of TNBC [5,71]. Two PARP inhibitors have been approved by the FDA and EMEA for patients with BRCA mutation-associated ABC: olaparib and talazoparib. In the OlympiAD trial [72], 302 women with previously treated HER2-negative ABC were randomised 2:1 to receive olaparib or chemotherapy of physicians’ choice (which did not include a platinum). Patients with TNBC (*n* = 150) were required to have received no more than two prior lines of chemotherapy. The primary outcome of PFS was met (HR 0.58, 95% CI 0.43–0.80, *p* < 0.001) [72] with an improvement in PFS from 4.2 months in the control arm to 7.0 months with olaparib. The benefit appeared to be most marked in the TNBC sub-group (HR 0.43, CI 0.29–0.63). Final analysis demonstrated a numerical but not statistically significant improvement in OS in the full study population (HR 0.90, CI 0.66–1.23) [37], likely influenced by more patients on the standard arm accessing PARP inhibitors and/or platinum-based chemotherapy as subsequent treatments. Sub-group analyses also demonstrated a trend towards improved OS in those treated earlier in their disease course [37], and further studies investigating olaparib in the first line are awaited.

The EMBRACA trial similarly randomised 431 patients with *BRCA*-mutated ABC to talazoparib or treatment of the physician’s choice (again not permitting platinum). EMBRACA also met its primary end point of improved PFS (median 8.6 vs. 5.6 months, HR 0.54, CI 0.41–0.71, *p* ≤ 0.001) [73] and reported a doubling of ORR (66.6% vs. 27.2%), but demonstrated no statistical benefit in OS (HR0.85, CI 0.67–1.07) [38]. Again, this may be explained by more patients in the control arm subsequently receiving a PARP inhibitor.

Both PARP inhibitors are associated with a favourable toxicity profile compared to standard chemotherapy, with the most frequent non-haematological side-effects of the PARP inhibitors being low-grade nausea and vomiting, which improved with treatment continuation. Haematological toxicities were the most frequent grade 3 events for both olaparib and talazoparib, including anaemia (16% and 39%) and neutropenia (9.3% and 21%) in the OlympiAD and EMBRACA [73] studies, respectively.

### 4.2. HER2 Mutations

HER2 protein overexpression or gene amplification, identified in 15–20% of breast cancers, has been used to classify breast cancer as HER2-positive or negative for over two decades. Rarer mutations in *HER2* are also tumourigenic [74] and occur in between 2 and 5% of all HR+/HER2-negative breast cancers but are not usually associated with HER2 over expression or gene amplification [75,76]. *HER2* mutations are rare in TNBC (1–2% [77,78]) but enriched in lobular breast cancer [79].

The HER2-targeted drug, neratinib combined with trastuzumab was evaluated in *HER2*-mutated cancers within the SUMMIT study [41] and included 18 patients with advanced TNBC. An ORR of 33% and a clinical benefit rate of 39% was reported [41]. Arguably, this provides a useful treatment option for the small group of women with *HER2*-mutated advanced TNBC. A selective HER2 TKI, tucatinib, has also undergone evaluation with trastuzumab in *HER2*-mutated cancers and results from the breast cancer cohort are awaited (NCT04579380).

### 4.3. AKT Pathway

The phosphatidylinositol 3-kinase (PI3K)/AKT pathway is frequently dysregulated in TNBC. Usually this is due to activating mutations in *PIK3CA* or *AKT1* as well as loss of function mutations in *PTEN* [80,81,82]. AKT is the central hub of a variety of signalling pathways that promote cell growth as well as invasion and migration of cells [83], the activation of which has been associated with a worse prognosis and resistance to treatment [84]. *AKT1* can be targeted clinically by the highly potent kinase inhibitor capivasertib. Within the UK PlasmaMATCH study, 11 of the 179 patients with TNBC had *AKT1* or *PTEN* mutations [77], six of whom were treated with capivasertib. No objective responses were seen in those with *PTEN* mutations, but in the *AKT1* mutant cohort, a response rate of 33.3% was observed. This was a heavily pre-treated population that may have influenced drug resistance; however, the results still suggest that AKT1 inhibition is a potentially useful treatment for patients with these rare sensitising mutations.

The combination of capivasertib with chemotherapy has demonstrated synergy in pre-clinical studies [85] and has been investigated in TNBC in the PAKT trial [39]. In this phase II study, patients with previously untreated locally advanced or metastatic TNBC were randomised to paclitaxel with capivasertib or placebo. The study met its primary end point of improved PFS (median 5.5 vs. 3.6 months, HR 0.64, CI 0.43–0.95) in the capivasertib group. In the sub-group of patients with *PIK3CA/AKT1/PTEN* alterations, median PFS was a more encouraging 9.3 months with capivasertib/paclitaxel vs. 3.7 months (HR 0.30, CI 0.11–0.79) with paclitaxel/placebo. Median OS was also improved in the capivasertib arm at 19.1 months vs. 12.6 months in the placebo arm (HR0.61 CI 0.37–0.99) and not reached in the pathway altered cohort. Similar results were reported from the phase 2 LOTUS study of another AKT1 inhibitor, ipatasertib, in combination with paclitaxel [86]: Median PFS in the pathway altered group (*n* = 42) was 9.0 vs. 4.9 months (HR 0.44, CI 0.2–0.99) and there was a numerical OS advantage to ipatasertib, although this did not reach statistical significance in either the ITT or the pathway altered populations [40]. No AKT inhibitor is currently licensed, but the phase III CAPItello290 trial of first-line capivasertib with paclitaxel is open to recruitment.

### 4.4. FGFR

Fibroblast growth receptors (FGFR) 1–4 constitute a family of tyrosine kinase receptors implicated in the pathogenesis of a variety of solid tumours, including breast cancer [87]. All four members of the FGFR family have been associated with specific features of breast cancer via diverse alterations, including amplifications, reported in 18% of breast cancers [88], activating mutations, and fusions. In TNBC *FGFR1* amplification is an independent negative prognostic factor [89] as well as conferring endocrine resistance in ER+ disease [90]. *FGFR2* amplification has also been associated with endocrine resistance [91] but is uncommon in breast cancer (2%). In pre-clinical models of TNBC, both FGFR3 and FGFR4 appear to have oncogenic driver roles [92,93]. Several FGFR inhibitors have reached clinical trials with early non-selective FGFR inhibitors being replaced with selective inhibitors targeting specific FGFR receptors. In the phase II basket study NCI-MATCH [94], 33% of patients in the cohort who received the FGFR inhibitor, AZD4547, had heavily pre-treated ABC. The majority of the breast cancer patients had *FGFR1* amplification and it is increasingly clear that this alteration is complex and likely encompasses other genes that might contribute to disease progression, which may in part explain the low ORR seen (8%).

The predominant interest in FGFR inhibition has centred on HR+ positive disease as they more frequently harbour *FGFR* alterations; however, cohort 2 of the phase II FOENIX study of futibatinib, (NCT04024436) specifically recruited patients with advanced TNBC and *FGFR2* amplification. All patients have received at least one prior line of chemotherapy for advanced disease and the results are awaited.

While targeting FGFR remains an interesting therapeutic strategy, more work is needed to establish the role of individual changes in the FGFR pathway and their role in response to targeted agents. Identifying biomarkers for the range of previously described alterations will be an important step towards appropriate patient selection. *FGFR2* amplification may represent a more accurate biomarker of response to FGFR inhibition than *FGFR1* due to its ability to induce FGFR “addiction” and subsequent PI3K and mTOR signalling-dependence on FGFR [95]. *FGFR2* amplification predicted response to the FGFR inhibitor AZD4547 and PD173074, both in vitro and in a translational clinical trial [95]. However, the level of amplification and heterogeneity of amplification within a tumour is likely to be important, with a higher copy number and homogenously amplified tumour prediction for better responses to treatment [95].

### 4.5. Androgen Receptor

The LAR subtype accounts for 15–20% of TNBC and has been associated with a more indolent course and more favourable phenotype than other subtypes [96]. IHC detection of the androgen receptor (AR) is a potential surrogate marker for this subset of TNBC, but although the LAR subtype has higher median AR expression by IHC [96], the degree of correlation between AR expression and LAR subtype is uncertain. Direct targeting of the AR has been disappointing in clinical practice, with only modest response rates reported with the AR antagonists enzalutamide [42] and bicalutamide [43], and the 17-alpha-hydroxylase inhibitor abiraterone in women with advanced TNBC, which is AR+ on IHC [44]: Response rates were typically less than 10% and median PFS below 3 months.

LAR-subtype cancers appear less immunogenic than basal TNBCs, suggesting they may have differential responses to current standard treatments. Combination studies with AR-targeted agents and the CDK4/6 inhibitor palbociclib (NCT02605486) or the *PIK3CA* inhibitor, alpelisib (NCT03207529) are attempting to improve the efficacy in this population and are currently underway. Palbociclib is also being investigated in combination with the anti-PD-L1 antibody avelumab in AR+ TNBC (NCT04360941).

### 4.6. Targeted Agents Summary

The identification of specific vulnerabilities within the molecular makeup of TNBC provides new targets for the clinical application of treatment combinations. It is likely that TNBC treatment algorithms will diverge further based on molecular subtyping in the coming years.

## 5. Future Directions

Circulating tumour DNA (ctDNA) assays, which are also known as liquid biopsies, have become a useful option for detecting actionable molecular changes in ABC, with several commercial assays now available. The potential for using ctDNA to predict risk of recurrence [97], response to treatment [98], and to monitor the evolution of new resistance mechanisms has been demonstrated in principle in ER-positive ABC [99,100] and is being tested prospectively in clinical trials (such as NCT04920708). In TNBC, ctDNA assays are similarly an option for detecting somatic *BRCA* and *HER2* mutations and TMB, but whether it can predict treatment failure early enough to allow intervention and ultimately change the outcome is unknown. In early TNBC, the presence of ctDNA following treatment is predictive of early recurrence [101], but the first study of its kind, C-TRAK, using ctDNA tracking in high-risk TNBC after completion of adjuvant therapies, failed to demonstrate benefit from pembrolizumab for patients with ctDNA relapse, at least in part, because the majority of patients already had demonstrable metastatic disease when imaged [102].

## 6. Conclusions

The term TNBC currently encompasses a diverse group of breast cancers that will only be optimally treated once the potential weaknesses that their distinct molecular features confer can be both identified and targeted. The introduction of ICI, the development of ADCs, and the identification of selected targeted therapies is finally broadening the treatment landscape for TNBC (Figure 1 and Figure 2), but survival still lags well behind either ER-positive or HER2-positive patients. Furthermore, almost all advanced cancers develop resistance to successive lines of systemic therapy and, therefore, future research needs to identify ways of overcoming this acquired resistance. This can potentially be achieved through evaluating combinations of ICI with other immune-modulating agents, targeted agents, and ADCs.

## Figures and Tables

**Table 2:** Phase 2/3 trials of ADCs and targeted agents including patients with TNBC.

Trial Name and Sample Size	Treatment Arms	Response Rate (%)	Median PFS/Months	Median OS/Months (ITT)
ADCs				
ASCENT [35] *n* = 468	Chemotherapy (Eribulin, vinorelbine, capecitabine or gemcitabine)	5	1.7	6.7
	Sacituzumab govitecan	35	5.6	12.1
DESTINY BREAST-04 [36] *n* = 63 with TNBC (ITT 557 HER2 Low)	Chemotherapy (capecitabine, eribulin, gemcitabine, paclitaxel or nab-paclitaxel)	16.3	5.4	8.3 (16.8)
	Trastuzumab deruxtecan	52.6	10.1	18.2 (23.4)
PARP Inhibitors				
OlympiAD [37] *n* = 150 TNBC (ITT = 302 BRCA mt)	Chemotherapy (capecitabine, eribulin or vinorelbine)	28.8	4.2	19.3
	Olaparib	59.9	7.0	19.6
EMBRACA [38] *n* = 190 TNBC (ITT = 431 BRCA mt)	Chemotherapy (capecitabine, eribulin, gemcitabine or vinorelbine)	27.2	5.6	19.5
	Talazoparib	67.6	8.6	22.3
AKT pathway targeted agents				
PAKT [39] *n* = 28 AKT pathway mutations (ITT *n* = 140) All TNBC	Chemotherapy (paclitaxel) + placebo	18.2 (28.8)	3.6 (3.6)	10.4 (12.6)
	Chemotherapy (paclitaxel) + Capivasertib	35.3 (34.8)	9.3 (5.5)	NR (19.1)
LOTUS [40] *n*= 42 AKT pathway mutations (ITT *n* = 124) All TNBC	Chemotherapy (paclitaxel) + placebo	26 (32)	4.9 (4.9)	22.1 (16.9)
	Chemotherapy (paclitaxel) + ipatasertib	48 (40)	9.0 (6.2)	25.8 (25.8)
HER2 mutation				
SUMMIT [41] (TNBC) *n* = 18 (ITT *n*= 18)	Neratinib + Fulvestrant + Trastuzumab	33.3 (46.2)	6.2	NR
AR targeted trials				
MDV3100-11 [42] *n* = 83 TNBC (ITT *n* = 118)	Enzalutamide 160 mg/day	6	2.9	12.7
NCT00468715 [43] *n* = 26	Bicalutamide 150 mg/day	0	12 weeks	NR
UCBG 12-1 [44] *n* = 34	Abiraterone 1000 mg/day	6.7	2.8	NR

BRCA mt = patients with BRCA1 or 2 gene mutations; PFS = progression free survival; OS = overall survival; ITT = intention to treat; NR = not reported; TNBC = triple negative breast cancer.

**Table 3:** Currently recruiting/recently accrued clinical trials in advanced TNBC.

Clinical Trials.gov Identifier; Trial Name	Patient Population	ADC	Target	Combination or Monotherapy	Phase	Planned (*n*)
**Combination studies of approved ADCs:**						
NCT04039230	Metastatic TNBC	Sacituzumab govitecan	TROP-2	Talozaparib	1/2	75
NCT04468061	Metastatic TNBC	Sacituzumab govitecan	TROP-2	Pembrolizumab	Randomised phase 2	110
NCT03424005 (MORPHEUS-TNBC)	Metastatic TNBC	Sacituzumab govitecan	TROP-2	Atezolizumab	1/2	242
NCT05374512 (TROPION-BREAST02)	Metastatic TNBC	Dato-DXd	TROP-2	Monotherapy	3	600
NCT04556773 (DESTINY-BREAST08)	HER2 low MBC	Trastuzumab deruxtecan	HER2	Chemotherapy/immunotherapy/hormone therapy	1b	139
NCT05382299 (ASCENT-03)	Metastatic TNBC	Sacituzumab govitecan	TROP-2	Monotherapy	3	540
NCT05382286 (ASCENT-04)	Metastatic TNBC	Sacituzumab govitecan	TROP-2	Pembrolizumab/chemotherapy	3	440
**Novel ADCs in development in breast cancer**						
NCT04742153	HER2-low MBC	MRG002	HER2	Monotherapy	2	66
NCT04152499	TNBC (and other solid tumours)	SKB264	TROP-2	Monotherapy	1/2	78
NCT04064359	CD205+ HER2-negative MBC (plus other solid tumours)	OBT076	CD205	Monotherapy	1	70
NCT03504488	TNBC (or NSCLC, or STS)	CAB-ROR2-ADC	ROR2	Monotherapy	1/2	120
NCT04300556	TNBC and other selected solid tumours	MORAb-202	Folate receptor alpha	Monotherapy	1/2	196
NCT03401385 TROPION-PANTUMOUR01)	TNBC and ER+ breast cancer (and NSCLC)	Datopotamab Detuxtecan,	TROP2	Monotherapy	1	770
NCT03742102	TNBC	Datopotamab Detuxtecan,	TROP2	Combination with Durvalumab	1/2	57
NCT04441099	TNBC and other solid tumours + sarcoma	NBE-002	ROR1	Monotherapy	1/2	100
NCT05498597	TNBC and other solid tumours	AMT-151	Folate receptor alpha	Monotherapy	1	30
NCT04699630	TNBC and other solid tumours	US-1402	HER3	Monotherapy	2	120
NCT05579366	TNBC and other solid tumours	PRO1184-001	Folate receptor alpha	Monotherapy	1/2	134
NCT03310957	Metastatic TNBC	Ladiratuzumab vedotin	LIV-1	Pembrolizumab	1/2	211
NCT05866432 (TUXEDO-2)	Metastatic TNBC with brain metastases	Datopotamab deruxtecan	TROP-2	Monotherapy	2	20
NCT05377996	TNBC and other solid tumours	XMT-1660	B7-H4	MONOTHERAPY	1	166
NCT04925284 (JEWEL-101)	TNBC and other solid tumours	XB002	Tissue factor	Monotherapy/with nivolumab/bevacizumab	1	561
NCT05208762	TNBC and other solid tumours	SGN-PD-L1V	PDL1	Monotherapy	1	315
NCT05194072	TNBC and other solid tumours	SGN-B7H4V	B7-H4	Monotherapy	1	400
NCT04225117	TNBC and other solid tumours	Enfortumab vedotin	Nectin-4	Monotherapy	2	288
NCT 02980341	HER3 Positive BC	Patritumab deruxtecan	HER3	Monotherapy	1/2	184
NCT04699630	TNBC and other breast cancer subtypes	Patritumab deruxtecan	HER3	Monotherapy	2	120
NCT05831878	Advanced HER2 low breast cancer	Disitimab vedotin	HER2	Monotherapy	N/a	36

TNBC = triple negative breast cancer, MBC = metastatic breast cancer, ADC = antibody drug conjugate, NSCLC = non-small cell lung cancer, ER+ = oestrogen receptor positive.
